# Bacteriophage Therapy in Selected Disease Entities: A Review of the Literature

**DOI:** 10.7759/cureus.92463

**Published:** 2025-09-16

**Authors:** Adam Dudek, Marcin Bursy, Wojciech Szkudlarek, Jan Linkiewicz, Piotr Starosta, Zbigniew Fabiszewski, Magdalena Grabinska

**Affiliations:** 1 General Practice, University Hospital in Poznan, Poznań, POL; 2 Medicine, Medical Center HCP, Poznań, POL; 3 Medical School, Poznan University of Medical Sciences, Poznań, POL; 4 General Practice, Independent Healthcare Facility in Oborniki, Oborniki, POL; 5 General Surgery, Independent Healthcare Facility in Oborniki, Oborniki, POL; 6 General Practice, Józef Struś Multispecialty Municipal Hospital, Poznań, POL

**Keywords:** antibiotics resistance, bacteriophages, bacteriophage therapy, chronic rhinosinusitis, infective endocarditis

## Abstract

Bacteriophage therapy is gaining increasing interest in the medical world due to the search for new treatments for bacterial infections in the era of antibiotic resistance. However, its use in the treatment of chronic rhinosinusitis (CRS) and infective endocarditis (IE) remains a challenge. Antimicrobial therapy with bacteriophages faces challenges such as the identification of the specific type of bacteriophage and the bacterium sensitive to it, the method of administration, and the safety of use. The aim of this paper is to review the available literature on the feasibility of bacteriophage therapy for CRS and IE, with a focus on its potential as an alternative or adjunctive approach to conventional antibiotic treatment. The analysis was based on articles available on the PubMed platform; original publications describing experimental trials investigating the potential efficacy of bacteriophages in the treatment of CRS and IE, published within the last 10 years, were selected for this review. Articles were searched using the following keywords: "phage therapy," "chronic rhinosinusitis," and "phage therapy infective endocarditis." A total of 54 articles were found, of which seven original papers were included in the review after reviewing the abstracts. Bacteriophage therapy has shown promising potential in the treatment of CRS and IE, but its clinical application remains limited by challenges such as the identification of susceptible bacterial strains, mode of administration, dosage, and safety of use. Future research must be done to unlock the full therapeutic potential of bacteriophage therapy for these indications.

## Introduction and background

Bacterial infections represent a major challenge in everyday medical practice. Since prehistoric times, mankind has actively sought ways of treating them, most often looking for possible curative actions among products of plant origin. For example, the Ancient Egyptians recognized the common garlic, Allium sativum, as a plant to help treat infectious diseases [1]. Modern research has shown that aqueous extracts of garlic have been shown to inhibit the in vitro growth of certain bacterial strains, including *Shigella* and *Salmonella* [2]. Nevertheless, despite often bold attempts at treatment, the efficacy of therapies for diseases caused by bacteria was relatively low; a breakthrough in this regard came with the discovery of penicillin by Alexander Fleming in 1928, which proved to be effective in treating bacterial infections [3]. As medicine continued to develop, the number of new antibiotics increased, giving doctors an effective tool to treat diseases caused by bacteria, thus enabling many lives to be saved.

Antibiotics are chemicals that are generally responsible for inhibiting bacterial cell division and/or causing cell death. This is done through a variety of biological mechanisms. For example, antibiotics in the β-lactam group exhibit antibacterial activity by inhibiting the synthesis of the bacterial cell wall, but they do not exhibit activity against eukaryotic cells, as these cells do not have a cell wall. Thus, antibiotics in this group only show selective action against bacterial cells [4]. The differences in structure and physiology between bacterial and eukaryotic cells also provide a choke point for the action of other antibiotics, limiting their action primarily to bacterial cells [3,4]. Unfortunately, the problem of microbial resistance to the antibiotics used by doctors is becoming increasingly prevalent in modern medical practice [5]. The reasons for the spread of this resistance are manifold, including the selection of natural strains of antibiotic-resistant bacteria through overuse or inappropriate therapy, uncontrolled use of antibiotics in the meat industry, genetic mutations in bacterial genomes resulting in the acquisition of antibiotic-resistant genes, the acquisition of these genes by bacterial cells through the process of conjugation and transduction, etc. [6,7]. In addition, the modern transport network connecting basically every corner of the world also contributes to the spread of antibiotic-resistant bacterial strains worldwide [8,9]. An additional problem is the slowdown in the search for new antibiotics; of the 20 companies that were involved in the development of new antibiotics in the 1980s, only five are still active [8]. This leads to very serious public health consequences, with an estimated 7,00,000 people currently dying each year from infections with antibiotic-resistant bacterial strains. This figure is expected to rise to millions of deaths per year by 2050 if the antibiotic situation remains unchanged [8]. As a result, the efforts of the medical community are nowadays focused on actively searching for alternative therapies for diseases caused by bacteria. A promising treatment for these infections is bacteriophage therapy [10].

The aim of this paper is to review the available literature describing the feasibility of bacteriophage therapy for chronic rhinosinusitis (CRS) and infective endocarditis (IE) as an alternative or adjunctive therapy to classical treatment with antibiotics. The analysis was based on articles available on the PubMed platform and original publications describing experimental trials investigating the potential efficacy of bacteriophages in the treatment of CRS and IE published within the last 10 years. The articles were selected for this review. Articles were searched using the following keywords: "phage therapy chronic rhinosinusitis" and "phage therapy infective endocarditis." A total of 54 articles were found, of which seven original papers were included in the review after reviewing the abstracts.

## Review

Bacteriophages

Bacteriophages (phages) are infectious agents belonging to viruses that infect bacterial cells. They are very widespread in the environment; it is estimated that there are ~107 phages in one milliliter of seawater. They are so common that they are also a natural part of the human microbiome [6,11]. These viruses are highly specific towards bacteria, attacking only bacterial cells; often only a specific species, a few are capable of attacking several types of bacteria, provided that the prokaryotes are phylogenetically closely related [11]. Phages multiply in bacterial cells in two ways. The first is the lysogenic cycle, in which the bacteriophage injects its genetic material into the bacterial cell, where it becomes integrated into the host genome and remains dormant as a prophage. The second possible course of infection is the lytic cycle, in which the phage genetic material is expressed in the bacterial cell, causing the formation of new phages and subsequent lysis of the bacterial cell. The lysogenic cycle can be converted into a lytic cycle through the expression of viral DNA integrated into the host DNA [6,11]. The majority of lytic phages active against human bacterial pathogens belong to the Caudovirales and Microviridae and are characterized by having genomes constructed from single- or double-stranded DNA [6]. An especially important feature of bacteriophages is their ability to act against biofilms, which are complex, structured bacterial communities encased in an extracellular matrix. Phages are capable of penetrating these protective layers through enzymatic degradation of the biofilm matrix, often via the production of depolymerases. In addition, their capacity to replicate within the biofilm environment allows them to gradually dismantle the structure from within, a task that most antibiotics cannot effectively accomplish [12,13]. Phages also demonstrate the unique advantage of self-amplification at the site of infection. Unlike conventional drugs, they increase in number as long as susceptible bacteria are present, thereby sustaining their antibacterial effect without repeated high dosing. This makes them particularly well-suited for localized infections, such as those in chronic rhinosinusitis or infective endocarditis [6,14].

While generally regarded as safe, bacteriophage therapy can interact with the host immune system. Lysis of large numbers of Gram-negative bacteria may result in the release of endotoxins, which requires careful clinical monitoring. Additionally, repeated exposure to phages may elicit an immune response, leading to the production of neutralizing antibodies that could reduce therapeutic efficacy during systemic treatment [11,15]. Overall, the mechanisms by which bacteriophages exert their effects underscore their potential as precise and dynamic antibacterial agents, especially in the face of growing antibiotic resistance.

Comparison of antibiotic and bacteriophage therapy

The first attempts to use bacteriophages to treat bacterial diseases took place more than a century ago. However, the growing problem of antibiotic resistance has renewed interest in the potential use of phages to treat bacterial infections, either when antibiotic therapy has failed or as an alternative to conventional antimicrobial treatment [6,14].

A comparison of antibiotic and bacteriophage therapy is shown in Table 1.

**Table 1:** Comparison of selected features of bacteriophage therapy and antibiotic therapy. [6,14]

Category	Phage therapy	Antibiotic therapy
Mechanism of action	Lysis of the bacterial cell by infection and lytic cycle	Disruption of key biological processes in the bacterial cell e.g., disruption of cell wall synthesis, DNA replication and DNA transcription
Specificity	High in relation to a specific type of bacterial microorganism	Depending on the type of antibiotic, broad or narrow, adversely affects the microbiome
Adaptability to evolving resistance	Co-evolution with the development of bacterial resistance	Unchanging, bacterial resistance limits the effectiveness of the therapy
Production	Relatively easy and inexpensive production, possibility to obtain phages from the environment	Well-established, the introduction of new drugs can be limited by the unfavorable ratio of costs incurred to potential gains limited by the rapid development of resistance and the restriction of the use of antibiotics
Effects on human cells	No direct cytotoxicity	May cause toxic effects, other side effects and allergic reactions
Mechanisms of bacterial resistance	Bacterial receptor mutations, CRISPR, etc.	Inactivating enzymes, efflux pumps etc.
Therapeutic challenges	It requires precise diagnosis and identification of the infecting bacterium, development of resistance. Still an experimental therapy	Microbiota composition disorders, allergic reactions and bacterial resistance

Bacteriophages in the treatment of chronic sinusitis

Sinusitis is a condition caused by simultaneous infection of the nasal mucosa and the mucosa lining the lumen of the paranasal sinuses; if the inflammation persists for more than 12 weeks, it meets the criteria for diagnosis of CRS [12,16,17]. CRS is a heterogeneous group of diseases that is highly prevalent, estimated to affect around 5-15% of the global population [13,18]. This disease is a serious health problem, causing a reduced quality of life for those affected. Despite this, its pathophysiology is not fully understood. Earlier models assumed that CRS develops when acute inflammation is not treated through impaired ventilation and drainage of sinus contents. However, it is now thought that the etiology of CRS is much more complex. It is assumed that disrupted interactions between the immune system and environmental factors, including infectious agents, are responsible for its development [17]. Patients affected by CRS most commonly report a feeling of a blocked nose, increased discharge from the nose or along the back wall of the throat, and deterioration of smell and taste. In addition, patients experience a spreading sensation in the sinus floor, sinus pain, cough, otalgia, fatigue, and sleep disturbances. The diagnosis of CRS is made on the basis of endoscopic examination, which reveals mucosal swelling, increased nasal cavity secretions, and mucosal polyps. In addition, a low-dose sinus CT scan may be considered if the picture on endoscopic examination is unclear. Olfactory tests and quality of life questionnaires may also be useful [17]. It is noteworthy that no studies have been published confirming the spontaneous cure of CRS [17]. Standard treatments for CRS include topical steroids, nasal cavity rinsing, antibiotics, and systemic corticosteroids. A challenge in the conservative treatment of CRS is the difficulty of removing the bacterial biofilm covering the sinus and nasal cavity mucosa and eradicating the multidrug-resistant bacterial strains that contribute to the development of CRS. Antibiotics show efficacy in treating acute inflammation, but unfortunately are no longer as effective in destroying the residual biofilm [15,19]. Surgical treatment is also applicable in the treatment of CRS, which is currently considered to be the most effective treatment for CRS, enabling a long-term therapeutic effect and even a permanent cure [17]. However, despite the relatively high success rate of surgical treatment, approximately 15-25% of patients require reoperation within 5-10 years after the initial surgery [17,20]. Among 50% of patients with refractory CRS and unsuccessful surgical treatment, a biofilm lining the mucosa of the nasal cavity and paranasal sinuses dominated by *Staphylococcus aureus *is observed [15,18]. Some researchers believe that colonization by *S. aureus* leads to the formation of superantigenic toxins that increase the local eosinophilic inflammatory reaction and influence the formation of polyps [21,22]. The attention of researchers is now turning to the role of bacteriophages in the treatment of CRS [12].

In the 2021 study ‘A randomized, double-blind, placebo-controlled study to investigate the use of bacteriophages in patients with chronic rhinosinusitis with nasal polyps,’ Dobrestov et al. investigated the effect of the preparation ‘Otophag,’ containing a mixture of 32 types of bacteriophages (showing colony-restrictive activity against bacterial colonies, i.e., *Bacteroides spp.*, *Escherichia coli spp.*, *Haemophilus influenzae spp.*, *Pseudomonas aeruginosa spp.*, *Staphylococcus aureus spp.*, etc.), administered to patients after surgical treatment of CRS with nasal polyps. The study was conducted on 40 CRS patients with nasal polyps aged 18-64 years undergoing endoscopic sinus surgery (ESS). Twenty subjects constituted the control group, which received an intranasal placebo after surgery, and 20 subjects constituted the study sample receiving intranasal ‘Otophag’; both groups were followed for 30 days. On the 10th and 13th days, swabs were taken from the nasal cavities of patients in both groups, and the number of bacterial colonies raised from the samples obtained was compared. After 10 days of Otophag application, a significantly reduced number of *S. aureus* Colony Forming Units (CFUs) was observed compared to the control sample; after 30 days of the experimental treatment, *S. aureus* remained absent from the samples taken from the patients in the test sample. In contrast, no statistically significant decrease in *S. aureus* CFUs was observed in the control sample [21].

The 2019 study ‘Exacerbations of Chronic Rhinosinusitis-Microbiology and Perspectives of Phage Therapy’ evaluated the feasibility of using bacteriophages to treat CRS. Microbiological samples were collected from 50 patients aged 25-80 years with CRS who had previously undergone an ESS procedure; only 10% of patients were identified as having bacterial grafts responsible for acute sinusitis [23]. Bacteria such as *Staphylococcus aureus*, *Pseudomonas aeruginosa*, *Escherichia coli*, *Klebsiella pneumoniae*, *Klebsiella oxytoca*, and *Acinetobacter baumannii* were identified in the majority of patients. Among the isolated strains, 28% showed features of antibiotic resistance [23]. The *S. aureus* isolate was coincubated with bacteriophages Pul/14/14256, Kr/6/1934, W/5/14256, and Kos/10/22119 at a concentration of 108 PFU/mL per phage. The results indicate that the bacteriophages used can be effective in up to 63% of CRS patients with colonization by *S. aureus* [23]. However, as the authors of the publication themselves point out, further research is required.

In a 2022 case report, Rodriguez et al. demonstrated the feasibility of successfully treating CRS with bacteriophages [24]. A 61-year-old woman diagnosed with sarcoidosis and diabetes had been suffering from CRS and recurrent middle ear infections caused by MRSA (methicillin-resistant *Staphylococcus aureus*) for several years. After repeated and ineffective antibiotic treatments and surgery, intravenous and topical phage therapy with SeMN68phi was decided upon, then, after partial improvement, therapy was continued with SaWIQ0456AØ phage administered intranasally and into the external ear canal. After two weeks, significant improvement and negative microbiological results were obtained. In 2021, a relapse occurred, which was again treated with SaWIQ0456AØ1 phage administered systemically and intranasally, achieving clinical improvement after one week and complete resolution of symptoms, persisting until at least March 2022 [24]. The case description indicates the possibility of using bacteriophages in the treatment of CRS, but further studies are required.

An important element in the research of new therapeutics is the evaluation of their safety. In 2019, the study ‘Safety and Tolerability of Bacteriophage Therapy for Chronic Rhinosinusitis Due to *Staphylococcus aureus*, a safety analysis of the use of bacteriophage AB-SA01 in nine patients with CRS refractory to conservative and surgical treatment was performed [15]. All patients showed the presence of *S. aureus* in cultures from their nasal cavities and paranasal sinuses. The median age of the patients was 45 years, and they were divided into three cohorts, with three patients in each cohort. Each cohort had bacteriophage AB-SA01 administered intranasally irrigations according to the following schedule: Cohort 1: bacteriophage at a concentration of 3 × 10⁸ plaque-forming units (PFU) for seven days; Cohort 2: 3 × 10⁸ PFU for 14 days; Cohort 3: 3 × 10⁹ PFU for 14 days. No serious adverse reactions or deaths were reported during the experiment. There were also no significant changes in physical examination, vital signs, temperature, or laboratory tests (except for one transient change in bicarbonate levels). During the study, six mild adverse reactions were reported in six patients, but all had resolved by the end of the study. Assessment of treatment efficacy was based on microbiological findings, sinus endoscopy, and symptom scales. Complete eradication of *S. aureus *was achieved in two of nine patients, with the remaining patients showing improvement in clinical symptoms and endoscopic findings [15]. The results indicate that CRS bacteriophage therapy was well tolerated among the subjects; however, due to the small group of patients included in the study and the lack of a control group, the results require further verification in other studies.

Bacteriophages in infective endocarditis

IE is a disease affecting the endocardium of the myocardium. Population-wise, there are 3-10/100,000 new cases each year. Despite ongoing advances in medical care, mortality remains high, i.e., up to 30% within 30 days of symptom onset [25]. In the epidemiology of IE, it has recently been observed that approximately 25-30% of the cases of this disease are associated with medical interventions such as those associated with the development of endovascular surgical treatment techniques [23]. Approximately 80-90% of IE cases have a bacterial etiology, with *S. aureus* being the most common causative agent of IE, accounting for approximately 26.6% of cases; the remaining cases are caused by *streptococci* and *enterococci* [25].

The clinical picture of IE includes high fever, night sweats, fatigue, loss of weight and appetite; cardiac arrhythmia and heart failure may also occur [26]. The diagnosis is made on the basis of the clinical picture, microbiological examination of blood samples, transthoracic echocardiography, which has a sensitivity of approximately 70% in detecting bacterial vegetations on the valves, and transesophageal echocardiography, which has a sensitivity of up to 90% in detecting these lesions [25,27]. The first line of treatment for IE is antibiotics; unfortunately, the increasingly emerging problem of antibiotic resistance is reducing their efficacy in the treatment of IE and forcing clinicians to look for new alternative treatments.

The study conducted in 2022 investigated the feasibility of using bacteriophage therapy to treat infective endocarditis, caused by *S. aureus*, in an animal model [28]. A rat model of infective endocarditis was used in the experiment. The animals were then divided into 4 groups, and treatment by central contact was initiated 6 hours after infection: in group 1, a phage cocktail containing vB_SauH_2002 (Herelleviridae) and Phage 66 (Podoviridae) was administered. Group 2 received flucloxacillin. Group 3 received a mixture containing bacteriophages and an antibiotic. Group 4 was the control group receiving saline. Then, after 30 h, bacterial burden and phage distribution were assessed [28]. A bacteriostatic effect was obtained in groups 1 and 2, while a synergistic effect was observed in group 3 receiving the phage and antibiotic preparation, achieving sterility of the endocardial vegetation in 12 of the 15 rats tested in this group [28]. The study showed that combining phage with an antibiotic for the treatment of *Staphylococcus aureus* endocarditis can significantly improve the efficacy of the therapy. However, phage therapy should not be used alone in vascular infections. Despite the promising results, further experimental studies, including human trials evaluating the efficacy and safety of the therapy, are needed [28].

In the 2023 study, Coyne et al. investigated the efficacy of antibiotic-phage therapy against a daptomycin-resistant strain of *Enterococcus faecium* in an ex vivo model [29]. This bacterial strain may be responsible for the development of IE resistant to standard treatment. Samples consisting of clots containing cultures of *E. faecium* incubated for 96 h at 37°C provided an ex vivo model of infective endocarditis. The bacterial strain tested was human blood-derived E. faecium R497, resistant to daptomycin (DAP MIC = 16 μg/mL), with LiaSR mutations associated with DAP resistance. The phages used in the study were bacteriophages NV-497 and NV-503-01 [18]. The ex-vivo model samples were divided into 12 groups, according to the scheme: Group 1: Daptomycin (DAP) alone; group 2: Ceftaroline (CPT) alone; group 3: Phage NV-497 alone; group 4: Phage NV-503-01 alone; group 5: Phage cocktail (NV-497 + NV-503-01); group 6: DAP + CPT; group 7: DAP + NV-497; group 8: DAP + phage cocktail; group 9: CPT + NV-497; group 10: CPT + phage cocktail; group 11: DAP + CPT + NV-497; group 12: DAP + CPT + phage cocktail. Dosing was as follows: DAP 10 mg/kg/day; CRT at 600 mg every 8 hours and phage cocktail (NV-497 + NV-503-01) at MOI (one bacteriophage per bacterial cell) = 1, administered every 24 h [29].

The best results were obtained in a sample containing daptomycin, ceftaroline, and a phage cocktail (NV-497 + NV-503-01). In this sample, bacterial counts fell from 8.77 to 3 log₁₀ CFU/g, a reduction of ~5.8 log. Only this combination gave a full bactericidal effect, and even a temporary resensitization to DAP was observed [29].

This publication showed that the concomitant use of bacteriophages and antibiotics can synergistically increase their mutual antimicrobial activity in the treatment of IE and may even lead to resensitization of the originally resistant strain to the antibiotic used. However, this topic needs to be further explored in studies [29].

A challenge in the treatment of IE is undoubtedly antibiotic-resistant bacterial strains, especially multidrug-resistant (MDR) and extremely resistant (XDR) strains. Kebriaeri et al. (2023) studied the efficacy of bacteriophage AB-SA01 (AmpliPhi Biosciences Corporation) against resistant strains of *P. aeruginosa *[30]. Two *P. aeruginosa* strains were used in the study: strain AR351 (XDR) and strain I0003-1 (MDR). Both strains were incubated in an ex vivo model simulating IE-developing vegetations consisting of human platelets, fibrinogen, and thrombin, which were infected with the aforementioned bacterial strains. Cell culture was carried out in vitro for each strain separately. Obtaining five groups of preparations each for *P. aeruginosa* strain AR51 with the following culture additions: Group 1 was the control group; group 2 was cultured with the addition of ciprofloxacin in monotherapy 400 mg every 12 hours; group 3 contained 3 types of phage from the AB-SA01 group, i.e., strain LL, strain EC, and strain 109; group 4 contained antibiotic, LL, and 109; and group 5 contained antibiotics and all 3 types of bacteriophages. For *P. aeruginosa* strain I0003-1, cultures were conducted in an analogous manner, with a difference in groups 4 and 5, which were conducted with the addition of ciprofloxacin, LL, and 109 for group 4 and ciprofloxacin, LL, EC, and 109 for group 5, respectively. The duration of the experiment was 96 hours [30]. The results showed that in the model in which both resistant bacterial strains were coincubated with the antibiotic and all types of phage, bacterial eradication to the limit of detection occurred. Accordingly, for *P. aeruginosa* strain AR351, the greatest reduction was observed: -5.65 log₁₀ CFU/g after 96 h (p < 0.001). In addition, a decrease in the limit of detection after 4 hours of incubation and no regrowth of bacterial colonies were reported throughout the experiment. For *P. aeruginosa* strain I0003-1, a decrease in bacterial colonies of -6.60 log₁₀ CFU/g was reported after 96 h (p < 0.001), with total bacterial eradication to the detection limit at 32 h. In this sample, no regrowth process was observed during the duration of the experiment.

This study indicates the potential efficacy of phage therapy as an adjunct to conventional antibiotic therapy for IE caused by resistant *P. aeruginosa* strains. However, due to the lack of confirmation of these results in an in vivo model and bearing in mind that the observation was only conducted for 96 hours, further investigation is required.

## Conclusions

The evolving antibiotic resistance of bacteria is an increasing problem in modern medicine, limiting the effectiveness of existing therapies. The available literature suggests that bacteriophages could be used as a new potential treatment for CRS and IE. Data from clinical trials reveal the efficacy of concomitant use of bacteriophages in combination therapy for the treatment of bacterial IE, compared to classical antimicrobial therapy. Bacteriophages also showed efficacy in the treatment of CRS caused by* S. aureus*, allowing, in some cases, the eradication of the pathogenic bacterium and a significant improvement in clinical status.

However, despite the promising results of the studies, further in-depth analysis on the use of bacteriophage therapy for these disease entities is required, involving much larger research groups and testing the efficacy and safety of these therapies. Some of the studies on this therapy for the treatment of CRS and IE have been conducted in vitro or on animal models; further research is required to verify the efficacy of these therapies in vivo in a human model. Nevertheless, bacteriophage therapy shows promising results as a potential treatment for CRS and IE.

## References

[1] Rauf A, Abu-Izneid T, Thiruvengadam M (2022). Garlic (allium sativum L.): its chemistry, nutritional composition, toxicity, and anticancer properties. Curr Top Med Chem.

[2] Bhatwalkar SB, Mondal R, Krishna SB (2021). Antibacterial properties of organosulfur compounds of garlic (allium sativum). Front Microbiol.

[3] Hutchings MI, Truman AW, Wilkinson B (2019). Antibiotics: past, present and future. Curr Opin Microbiol.

[4] Lima LM, Silva BN, Barbosa G, Barreiro EJ (2020). β-lactam antibiotics: An overview from a medicinal chemistry perspective. Eur J Med Chem.

[5] Huemer M, Mairpady Shambat S (2020). Antibiotic resistance and persistence-Implications for human health and treatment perspectives. EMBO Rep.

[6] Gordillo Altamirano FL, Barr JJ (2019). Phage therapy in the postantibiotic era. Clin Microbiol Rev.

[7] Munita JM, Arias CA (2016). Mechanisms of antibiotic resistance. Microbiol Spectr.

[8] Uddin TM, Chakraborty AJ, Khusro A (2021). Antibiotic resistance in microbes: History, mechanisms, therapeutic strategies and future prospects. J Infect Public Health.

[9] Larsson DG, Flach CF (2022). Antibiotic resistance in the environment. Nat Rev Microbiol.

[10] Prazak J, Iten M, Cameron DR (2019). Bacteriophages improve outcomes in experimental Staphylococcus aureus ventilator-associated pneumonia. Am J Respir Crit Care Med.

[11] Hatfull GF, Dedrick RM, Schooley RT (2022). Phage therapy for antibiotic-resistant bacterial infections. Annu Rev Med.

[12] Fong SA, Drilling A, Morales S (2017). Activity of bacteriophages in removing biofilms of Pseudomonas aeruginosa isolates from chronic rhinosinusitis patients. Front Cell Infect Microbiol.

[13] Łusiak-Szelachowska M, Weber-Dąbrowska B, Żaczek M, Górski A (2021). Anti-biofilm activity of bacteriophages and lysins in chronic rhinosinusitis. Acta Virol.

[14] Tagliaferri TL, Jansen M, Horz HP (2019). Fighting pathogenic bacteria on two fronts: phages and antibiotics as combined strategy. Front Cell Infect Microbiol.

[15] Ooi ML, Drilling AJ, Morales S (2019). Safety and tolerability of bacteriophage therapy for chronic rhinosinusitis due to Staphylococcus aureus. JAMA Otolaryngol Head Neck Surg.

[16] Rhinosinusitis. Wikipedia (2025). Wikipedia: Rhinosinusitis. https://en.wikipedia.org/wiki/Rinosinusitis.

[17] Hildenbrand T, Milger-Kneidinger K, Baumann I, Weber R (2024). The diagnosis and treatment of chronic rhinosinusitis. Dtsch Arztebl Int.

[18] Szaleniec J, Górski A, Szaleniec M (2017). Can phage therapy solve the problem of recalcitrant chronic rhinosinusitis?. Future Microbiol.

[19] Speck PG, Wormald PJ (2018). Is phage therapy suitable for treating chronic sinusitis Staphylococcus aureus infection?. Future Microbiol.

[20] Tan NC, Psaltis AJ (2020). Latest developments on topical therapies in chronic rhinosinusitis. Curr Opin Otolaryngol Head Neck Surg.

[21] Dobretsov KG, Kolenchukova O, Sipkin A (2021). A randomized, double-blind, placebo- -controlled study to investigate the use of bacteriophages in patients with chronic rhinosinusitis with nasal polyps. Otolaryngol Pol.

[22] Drilling AJ, Ooi ML, Miljkovic D (2017). Long-term safety of topical bacteriophage application to the frontal sinus region. Front Cell Infect Microbiol.

[23] Szaleniec J, Gibała A, Pobiega M (2019). Exacerbations of chronic rhinosinusitis-microbiology and perspectives of phage therapy. Antibiotics (Basel).

[24] Rodriguez JM, Woodworth BA, Horne B (2022). Case Report: successful use of phage therapy in refractory MRSA chronic rhinosinusitis. Int J Infect Dis.

[25] Rajani R, Klein JL (2020). Infective endocarditis: A contemporary update. Clin Med (Lond).

[26] Long B, Koyfman A (2018). Infectious endocarditis: An update for emergency clinicians. Am J Emerg Med.

[27] El-Dalati S, Cronin D, Shea M (2020). Clinical practice update on infectious endocarditis. Am J Med.

[28] Save J, Que YA, Entenza JM (2022). Bacteriophages combined with subtherapeutic doses of flucloxacillin act synergistically against Staphylococcus aureus experimental infective endocarditis. J Am Heart Assoc.

[29] Kunz Coyne AJ, Stamper K, El Ghali A (2023). Phage-antibiotic cocktail rescues daptomycin and phage susceptibility against daptomycin-nonsusceptible Enterococcus faecium in a simulated endocardial vegetation ex vivo model. Microbiol Spectr.

[30] El Ghali A, Stamper K, Kunz Coyne AJ (2023). Ciprofloxacin in combination with bacteriophage cocktails against multi-drug resistant Pseudomonas aeruginosa in ex vivo simulated endocardial vegetation models. Antimicrob Agents Chemother.

